# Hydrogen-Mediated Noncovalent Interactions in Solids: What Can NMR Crystallography Tell About?

**DOI:** 10.3390/molecules25163757

**Published:** 2020-08-18

**Authors:** Ioana Georgeta Grosu, Xenia Filip, Maria O. Miclăuș, Claudiu Filip

**Affiliations:** National Institute for R&D of Isotopic and Molecular Technologies, 400293 Cluj, Romania; ioana.grosu@itim-cj.ro (I.G.G.); xenia.filip@itim-cj.ro (X.F.); maria.miclaus@itim-cj.ro (M.O.M.)

**Keywords:** NMR crystallography, noncovalent interactions, hydrogen bonds, proton transfer

## Abstract

Hydrogen atoms play a crucial role in the aggregation of organic (bio)molecules through diverse number of noncovalent interactions that they mediate, such as electrostatic in proton transfer systems, hydrogen bonding, and CH–π interactions, to mention only the most prominent. To identify and adequately describe such low-energy interactions, increasingly sensitive methods have been developed over time, among which quantum chemical computations have witnessed impressive advances in recent years. For reaching the present state-of-the-art, computations had to rely on a pool of relevant experimental data, needed at least for validation, if not also for other purposes. In the case of molecular crystals, the best illustration for the synergy between computations and experiment is given by the so-called NMR crystallography approach. Originally designed to increase the confidence level in crystal structure determination of organic compounds from powders, NMR crystallography is able now to offer also a wealth of information regarding the noncovalent interactions that drive molecules to pack in a given crystalline pattern or another. This is particularly true for the noncovalent interactions which depend on the exact location of labile hydrogen atoms in the system: in such cases, NMR crystallography represents a valuable characterization tool, in some cases complementing even the standard single-crystal X-ray diffraction technique. A concise introduction in the field is made in this mini-review, which is aimed at providing a comprehensive picture with respect to the current accuracy level reached by NMR crystallography in the characterization of hydrogen-mediated noncovalent interactions in organic solids. Different types of practical applications are illustrated with the example of molecular crystals studied by our research group, but references to other representative developments reported in the literature are also made. By summarizing the major concepts and methodological progresses, the present work is also intended to be a guide to the practical potential of this relatively recent analytical tool for the scientists working in areas where crystal engineering represents the main approach for rational design of novel materials.

## 1. Introduction

The interest of chemists and scientists has been focused in the past century on the access to robust compounds with strong covalent bonds between atoms that exhibit high stability in environmental and harsh chemical conditions. The accelerated development of supramolecular chemistry and thus understanding chemistry beyond the covalent bond started in 1987, when Lehn, Cram, and Pederson were rewarded with the Nobel Prize for their contributions to the definition and elaboration of the principles of this new field. This has balanced the interest of scientists towards the intermolecular interactions and the properties of materials and compounds generated by the secondary (intermolecular) contacts between molecules [1,2,3].

Molecular interactions, also known as noncovalent or intermolecular interactions, are attractive or repulsive forces between molecules and between non-bonded atoms. Molecules can interact with each other in diverse number of ways, and thus intermolecular interactions play an essential role in many areas, like structural chemistry, self-assembly, supramolecular chemistry, biochemistry, drug design, protein folding, material science, and separations. The noncovalent self-assembly of molecules has led to spectacular supramolecular architectures and can induce exciting properties connected to the recognition of specific guests by designed hosts [4,5], self-sorting processes [6,7,8], or the selectivity of chemical reactions. [9,10,11]. Besides the classical hydrogen bonds, ubiquitous in nature and largely used as secondary interaction platform [12,13,14,15,16,17,18] for engineering advanced organic materials, other contacts, such as halogen bonds [19,20,21,22,23,24,25], hydrophobic contacts [26,27,28], π-donor–π-acceptor complexes [29,30,31], and interactions with anions [32,33,34,35], became powerful driving forces in the access not only to interesting supramolecular assemblies but also to compounds and materials with targeted properties/functionalities.

Despite the multitude of investigated contacts among molecules, the hydrogen bonds are still in the top of the interest for the design and fabrication of supramolecular architectures and rational design of systems for targeted applications. Nishio [36] classified these bonds in four categories corresponding to the classification of acids (A) and bases (B) in soft (S) and hard (H) ones and to their combinations. The four categories are HA/HB, HA/SB, SA/HB, and SA/SB. The classic interactions of OH and NH groups, with electronegative atoms (O, N), serve as example for the first category (HA/HB). The other contacts are of lower energies. The XH–π contacts belong to the HA/SB category, while C-H–X interactions are considered as example for the SA/HB type of contacts. The C-H–π contacts play in many cases an important role for the formation of supramolecular aggregates and they belong to the fourth category of hydrogen bonds involving soft acids and soft bases (SA/SB) [37]. Hydrogen bond donor character increases with acidity, while the acceptor character increases with basicity; multiple hydrogen acceptors can interact with a single donor, based on steric and geometric criteria, and they can be bifurcated (three center) or trifurcated (four center) [38].

Compared to covalent and ionic bonds, hydrogen bonds are weak interactions, which can be switched on and off with low energies and have a reversible character [39,40]. Therefore, they play a key role in biochemical processes occurring in aqueous media. Hydrogen bonds are responsible for a variety of recognition processes like enzyme–substrate biding and interactions between small biomolecules—ligands and proteins. These processes are so robust due to the reversible character of the hydrogen bonds and rely upon the formation and rupture of the bonds using water as medium for proton and/or electron transfer [41,42,43]. Hydrogen bonds are fundamental in biological systems also due to their role in stabilizing the structure of major building blocks of living organisms, like polypeptides/proteins [44] and DNA/RNA [45], where amine–carbonyl hydrogen bonds dominate, or polysaccharides biopolymers such as cellulose and chitin [46], where hydroxyls are those involved in hydrogen bonding.

As noncovalent interactions are so frequently found in nature, being determining factors for many structural, functional, and biological features, they also attracted scientists’ attention for their involvement in the rational design and synthesis of novel materials with desired/targeted physical and chemical properties. When the engineered systems/materials are crystalline, they fall in the already matured field of crystal engineering. A key concept introduced in this field is that of supramolecular synthon. Hydrogen bonds are directional; therefore, successful formation of hydrogen bonds between two molecules involves designing/choosing appropriate donor and acceptor moieties capable to engage in the interaction. The term supramolecular synthon has been proposed by Desiraju: “supramolecular synthons are structural units within supermolecules which can be formed and/or assembled by known or conceivable synthetic operations involving intermolecular interactions” [47]. The synthons can exist as homosynthons which contain identical functional groups with molecular complementarity like carboxylic acids or amides dimers [48], or heterosynthons that are composed on different but complementary functional groups. Heterosynthons are often based on O–H⋯N interactions, including carboxylic acid⋯⋯amide dimers and carboxylic acid⋯⋯aromatic nitrogen but also C–H⋯⋯N and C–H⋯⋯O, which are weaker interactions [49,50].

Multicomponent systems, like salts and co-crystals, also rely on the understanding of molecular recognition processes and complementary synthons where hydrogen bonding interactions with or without the transfer of hydrogen atoms play a key role in stabilizing the final structure. The cocrystallization process occurs through the direct self-assembly of different components, without covalent chemical modifications of the constituents [51]. Since their discovery, cocrystals have quickly attracted attention in pharmaceutical sciences, due to the fact that they offer the opportunity to modify properties of pharmaceutical compounds like solubility, stability, bioavailability, as it is well known that one of the major difficulties when developing new drug products is the poor oral bioavailability of the active pharmaceutical ingredient (API). Pharmaceutical cocrystals are crystalline solids composed of two (or more) neutral molecules, of which one is the API, and the second component is pharmaceutically accepted co-former (listed on the US Food and Drug Administration’s Generally Recognized as Safe-GRAS) [52,53]. Rational design of pharmaceutical cocrystals can be performed taking into account the possibility of hydrogen bonds formation between the API and the co-former, the use of complementary synthons. 

The hydrogen-mediated formation of multicomponent systems from supramolecular aggregates of biological interest to engineered crystalline organic materials can be nowadays studied with unprecedented precision by both, analytical and computational methods. Diffraction techniques are still playing the key role in the characterization of such systems via the determined crystal structures: the amount and quality of the extracted information, for instance, the exact geometry of the formed hydrogen bonding networks, depend on the precision with which the involved hydrogen atoms can be spatially confined based on such structures. However, the requirement here is that the compound can crystallize. Disordered/amorphous (bio)organic solids can instead be investigated only by spectroscopic techniques like solid-state NMR, FT-IR, or Raman. 

Neutron diffraction provides the best accuracy in hydrogen atoms localization [54], but this is not a common laboratory technique. Therefore, its use is limited to studies of more fundamental character, for instance the “migration” of a shared hydrogen atom between donor and acceptor sites in the so-called salt–cocrystal continuum [55]. Alternatively, most of the high-quality crystal structures of organic compounds are reported from single-crystal X-ray diffraction (XRD), because it is routinely available in conventional laboratories and the accuracy of locating non-hydrogen atoms approaches that of neutron diffraction. In addition, the systematically too short C(N,O, …)-H bond lengths determined by XRD can be compensated for by placing in the final structure the hydrogen atoms to standard neutron diffraction distances to the bonded hetero-atoms [54,56]. Such corrections are needed to account for the different ways that neutrons and X-rays are scattered by hydrogen atoms: the former interact with the well localized protons, whereas the later with the rather delocalized electron orbital surrounding the proton. Single-crystal XDR also suffers from inherent limitations, so that it cannot be generally applied to all the organic crystalline materials. The most prominent is the poor quality of the crystals that can be grown for many compounds, in terms of crystallite size, lattice defects, or the presence of structural/dynamic disorder. Consequently, powder techniques have emerged as promising alternatives, motivated primarily by numerous methodological advances, but also by practical demands especially from the pharmaceutical industry, for the development of fast crystal structure determination tools. With the advent of direct space search algorithms, powder X-ray diffraction (PXRD) has become increasingly popular for crystal structure determination of organic compounds from microcrystalline powders [57]. Drawbacks here occur from the reduced amount of information that can be extracted from 1D diffraction patterns recorded on powders, which is generally insufficient to constrain atomic positions with accuracy levels close to that characteristic to single crystal determined structures. This limits the complexity of the systems, measured through the number of flexible molecular degrees of freedom, like position within the unit cell and unconstrained torsion angles, which can be tackled by PXRD. As the complexity of the molecules in the lattice increases, the accuracy as well as the confidence level of the obtained structure solution(s) drop significantly [57]. Besides this, PXRD suffers from being insensitive to low weight atoms, particularly hydrogen, which directly relates to topic of the present work. As such, there has been a continuous search for additional sources of complementary structural information, including the precise location of hydrogen atoms, to be combined with the results of PXRD data analysis and provide in the end improved crystal structure models, ideally approaching the accuracy and confidence level achievable on single crystals.

Starting with the late nineties, such an approach that combines PXRD with solid-state Nuclear Magnetic Resonance (ss-NMR) spectroscopy, named NMR crystallography [58], has emerged as a new and powerful structural characterization tool on powders. More than three decades of continuous advances have contributed to reaching the present maturity level, which allows NMR crystallography to be employed not only for routine studies, but also in many spectacular applications. In this context, the structural determination of a complex molecular system with accuracy characteristic to single-crystal X-Ray diffraction [59], the re-evaluation followed by the correction of some older single-crystal structures in the Cambridge Crystallographic Database (CCD) [60], or the in depth study of an organic compound with six independent molecules in the asymmetric unit [61], are illustrative examples for the state-of-the-art reached in the field. NMR crystallography exploits the fact that local structural details are probed with the highest sensitivity by ss-NMR, whereas PXRD is very accurate in detecting long range ordering and crystal symmetries. Combining the two experimental techniques with first-principles quantum mechanical calculations makes the whole approach more powerful for structural characterization at supramolecular scale by correlating the computed parameters with ss-NMR and XRPD observables (chemical shifts, intra- and intermolecular distances, crystal packing patterns, etc.). 

The sensitivity of ss-NMR chemical shifts to both intra- and intermolecular structural parameters, thus also to crystal packing, is essential for the most of the NMR crystallography applications and can be exploited by comparing experimental values extracted from ^13^C/^15^N/^1^H ss-NMR spectra with theoretical values calculated on the proposed crystal structure model(s). However, as a direct interpretation of ^13^C/^15^N/^1^H chemical shifts in terms of well-defined structural features is excluded due to the interplay of multiple factors [58], the practical use of such an approach relied on the development of adequate computational methods. Computations based on Coupled Cluster Theory have the advantage of high accuracy, but their use in organic crystalline systems soon turned out to be impractical due to the poor scaling with the number of the considered orbitals. Years of intensive studies have demonstrated that, alternatively, the Density Functional Theory (DFT)-based approaches provide the best compromise between high accuracy and computation time. Particularly, the combination of the GIPAW method [62] with the PBE exchange-correlation functionals [63], the Monkhorst–Pack sampling grid [64], and the use of ultra-soft pseudopotential [65] within the CASTEP implementation [66] has “imposed” itself as a standard procedure in NMR crystallography, judging mainly from the number of published results, if not also by the reported accuracy. 

A detailed description of the methodological progresses in the field of NMR crystallography and of its numerous practical applications to a variety of crystalline organic systems can be found in excellent review articles [58,67,68,69,70]. Therefore, in this mini-review we narrow the perspective and focus the presentation only on recent developments leading to enhanced ability of NMR crystallography to constrain hydrogen atom positions with increasing accuracy in the determined crystal structures. For this purpose, crystalline organic systems investigated by our group are taken as a basis for illustrating the specific procedures employed under different circumstances for enhancing the chances to determine the real position of labile hydrogens. The previous results are re-interpreted under the common goal of finding the most suitable methods to overcome the difficulties characteristic to the nature of the investigated molecular crystal, and finally reach the desired accuracy level. For powders, they refer to uncertainties in the determined (multiple) crystal structure solutions, whereas for single crystals difficulties may arise from increased lattice disorder around the hydrogen atom of interest. The examples presented here clearly demonstrate that NMR crystallography has a great potential to become an indispensable analytical tool for identifying and characterization of hydrogen-mediated noncovalent interactions via its increasing accuracy in spatial proton localization.

## 2. How Accurate Can Hydrogen Atoms Position Be Constrained by NMR Crystallography?

**Figure 1:**
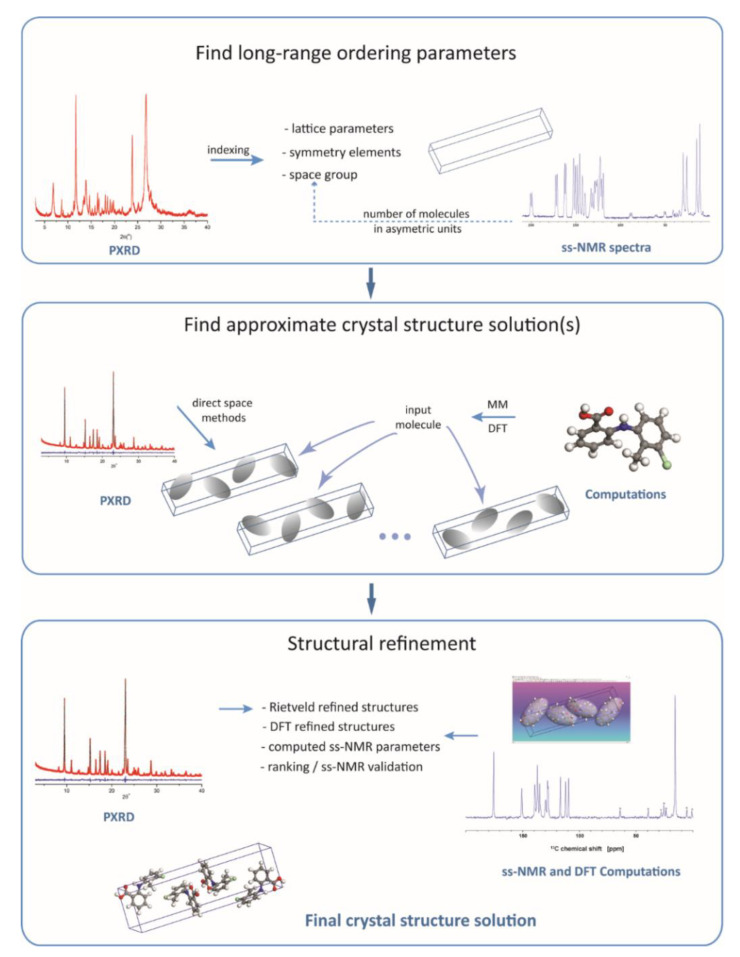
Schematic representation of the workflow used in an NMR crystallography approach to crystal structure determination.

The most general scheme for an NMR crystallography approach to crystal structure determination of organic compounds is shown in Figure 1. The three major stages of the process, namely, (i) find the characteristic long-range ordering parameters, (ii) search for all the plausible structure solutions, and (iii) drive the search towards the most realistic structural model by final refinement of the atomic positions, are in principle the same as in standard powder X-Ray crystal structure determination protocols. The difference is that experimental ss-NMR data and information generated by quantum chemical computations are also actively employed in crystal structure determination by adding extra steps to the whole process, which are intercalated among the steps that would have been normally followed when relying on diffraction data only. 

The first stage of the workflow, depicted in Figure 1, retrieves the unit cell parameters and symmetry elements. This is done by diffraction pattern indexing and space group determination based on the systematic absence of certain (h k l) reflections in the pattern. The crystal lattice parameters are independent of the inner structure of the “objects” (molecules, in our case) within the unit cell: they only define the rules according to which these objects are arranged to fill in the space. As the diffraction techniques show the highest sensitivity exactly to such long-range ordering parameters, this first stage is generally performed by employing specific X-ray data analysis procedures, with no direct contributions from ss-NMR, or quantum chemistry computations; although, some exceptions have been reported. For instance, using the concept of the Wyckoff spectrum [71], complementary information extracted by ^31^P ss-NMR was used to unambiguously determine the space group in various phosphorus inorganic compounds [72,73,74]. The procedure reported there, however, does not apply also to organic molecules, for which indexing and space group determination is performed solely by diffraction methods. 

The accuracy with which the crystal lattice parameters are determined depends primarily on the quality of the sample, like the crystallinity degree and the presence or not of static/dynamic disorder within the lattice. In the case of high-quality samples, these parameters can be constrained very accurately from the X-ray data analysis, so that there is no need for further refinement. At the other extreme, that is, powdered samples with poor crystallinity and also with a certain degree of lattice disorder, it is quite likely that the crystal lattice parameters cannot be determined with the best accuracy. Here, the NMR crystallography approach may indirectly contribute to their improvement at later stages in the structural determination process. In the case of unit cell dimensions and angles, the usual approach is to include these parameters together with other relevant molecular degrees of freedom in the DFT geometry optimization of the proposed crystal structure solutions. This often resulted in non-negligible corrections of the unit cell parameters with respect to their values determined from the diffraction pattern indexing [75]. Low-quality PXRD patterns may also lead to multiple space groups during the search of the symmetry elements. In such cases, the number of molecules in the asymmetric unit, which can be readily extracted from ^13^C ss-NMR spectra, represents valuable information that can reduce ambiguity. Knowing this number, the space groups which generate crystals with density values outside the characteristic range for organic solids, ρ = 1.1–1.9 g/cm^3^, are excluded from the further analysis.

The second stage in the crystal structure determination (Figure 1) includes a set of operations applied with the purpose of finding an approximate structure solution. The operations refer only to the variable structural degrees of freedom, generally position in the unit cell and flexible torsion angles of the molecule(s), whereas bond angles, bond lengths, and even some torsion angles, like those in aromatic rings, are kept fixed to their chemically realistic values. Limiting the number of variable parameters to be optimized greatly simplifies the search process, which is important in the case of molecules with many flexible torsion angles but, of course, this comes at the expense of losing accuracy. To some extent, accuracy can be improved by employing first principles quantum chemical calculations to generate the starting molecular structure, instead of simplistic molecular mechanics approaches. 

The approximate solution found at this stage represents a model which, up to the uncertainties determined by the imposed constraints, has the closest possible structure with respect to the actual crystal structure of the compound. In essence, the procedure consists of fitting the experimental X-ray pattern with the simulated patterns of a large number of trial crystal structures generated by various search algorithms [57] while retaining in the end as an approximate solution the structure with the best fit factor, the so called R_wp_ figure of merit. As previously, it is mainly performed by specific means of diffraction data analysis, with only a few cases being reported when ss-NMR was employed in the process [76]. These cases generally refer to introducing additional structural constraints, mostly inter-atomic distances [77] that can be reliably determined from experimental ss-NMR data.

Once an approximate solution has been determined (often, multiple realistic solutions can be found), this is taken as input structure for the third stage, the final refinement stage. There are at least two refinement steps usually performed here, depending on the experimental or theoretical data defining the cost function. First, the Rietveld refinement is applied [57], which basically represents a more rigorous and accurate fitting of the diffraction pattern by also including some of the structural parameters kept fixed previously, until the lowest R_wp_ value is reached. The output of Rietveld analysis is further refined in the second step, generally by using DFT geometry optimization methods, but other first principles quantum chemistry computations have been reported as well [78]. This produces a relaxed structure with respect to the forces acting upon each atom, which is used next for a final analysis aimed at assessing its closeness to the real crystal structure of the compound. The analysis is centered on the comparison between the calculated and experimentally measured ss-NMR chemical shift values, but it often includes also other steps: (i) consistency check of the generated crystal packing patterns and the associated noncovalent interactions, especially those involving hydrogen atoms; (ii) ranking of the final crystal structure models with respect to the lattice energy and ss-NMR fit parameters when working with multiple structure solutions; and (iii) iterative refinements with the purpose of improving the model from the perspective of simultaneously fitting diffraction- and ss-NMR data.

Hydrogen atoms enter this general scheme at the beginning of stage two, when they are placed at the most chemically realistic positions relative to the bonded heteroatoms in the starting molecular structures. These relative positions are not changed during the search for the approximate crystal structure solution and Rietveld refinement, i.e., when fitting the X-ray diffraction pattern, because the diffraction line profiles are almost insensitive to the exact location of a given hydrogen atom out of probably tens of other hydrogens in the molecule. By contrast, the measured ss-NMR parameters are mostly sensitive to the local structure, for instance very small changes in the position of a labile hydrogen atom may have significant effects upon both, its own ^1^H chemical shift, and the donor/acceptor heteroatom chemical shift (^15^N, ^17^O, etc.) values. Thus, the power of the NMR crystallography approach resides in the fact that the ss-NMR parameters can be generally measured with chemical site resolution, which allows to monitor the effects of geometry optimization upon each individual atom. In principle, the DFT optimized Rietveld model can be reported as the final crystal structure solution, but using an additional set of complementary experimental data with high sensitivity to local structural changes, like the ss-NMR chemical shifts, may significantly increase the confidence level in the crystal structure determination process. 

As described in the introductory section, the hydrogen mediated noncovalent interactions play a crucial role in the aggregation of organic (bio)molecules, from living organisms, to materials and pharmaceuticals. The type, strengths, and directionality of these interactions depend primarily on the exact location of the hydrogen atom relative to the interacting atoms in the molecule, like donor–acceptor pairs, phenyl rings, etc. Confining the discussion to organic crystalline phase, it is obvious from the above brief presentation that, with the emergence of NMR crystallography, hydrogen atoms can be constrained with increasing accuracy from the determined crystal structure also for powdered compounds, without the requirement to grow them as single crystals. Consequently, also the possibility for better characterization of the induced intermolecular interactions is extended to a wider range of organic crystalline materials. This is beneficial for adjusting materials properties in a crystal engineering approach, as the determination of the right balance among various noncovalent interactions represents the condition for further properties improvement, for instance, by exploiting the concept of supramolecular synthons.

Although a general logical flow, like that illustrated in Figure 1, can be identified in the crystal structure determination by NMR crystallography, there are still many details in the analysis which have to be treated on a case by case basis. This should be definitely taken into account when an answer to the question stated in the title of the section is attempted. In practice, there are two major factors influencing the accuracy with which the hydrogen atoms can be located. From computational side, the position of the hydrogen atoms in the crystal structure solution is finally established after performing DFT geometry optimization of the model obtained from the PXRD data analysis. Problems may occur here when the PXRD structure is relatively far away from the real crystal structure of the compound; in such cases, the risk of having the computed structure trapped in a local minimum on the potential energy surface, thus still far from the real structure, is not negligible. Another aspect refers to the approximations included in the simulation algorithms: from this perspective, the extensive study of the temperature effects [79] or the development of the dispersion corrected functionals [80] are examples of recent advances, which increase the confidence level in the DFT geometry optimized crystal structures. From experimental side, the important factor is the sensitivity of the measured ss-NMR parameters relative to the desired structural features. ^13^C, ^15^N, and ^1^H chemical shift values are always taken into account in an NMR crystallography analysis due to their dependence on the intermolecular arrangements. Referring to the problem of the labile protons location, the dependence of the ^1^H chemical shift value on the relative position of the shared hydrogen atom with respect to the donor and acceptor atoms [81], or the high sensitivity of ^15^N chemical shift to proton transfer, are valuable probes, often exploited in practice [82]. Besides chemical shifts, other ss-NMR parameters like dipolar couplings used to determine distances between selected nuclei [83] or hydrogen-mediated J-couplings have been also shown as promising ss-NMR parameters that could increase the accuracy in structural determination by NMR crystallography.

The progresses witnessed by NMR crystallography over the last two decades offer now the possibility of extending structural determination from powders to virtually any kind of crystalline organic solids, with accuracy levels approaching in some cases that of single-crystal X-Ray diffraction. This covers also the problem of better locating labile hydrogen atoms, with real benefits for crystal engineering approaches aimed at developing materials with tailored functionalities based on the directional and reversible character of hydrogen bonding. Although a precision limit that could be generally guaranteed in any case is not possible to be defined, the numerous examples in the literature [67], including the systems investigated in our research group that are discussed in more details below [59,82,84,85,86,87], clearly illustrates the state-of-the-art reached by NMR crystallography in characterizing hydrogen-mediated noncovalent interactions. In fact, as most of these studies reveal, the power of this technique comes from the fact that potential ambiguities of the PXRD structural models, or limitations in the DFT geometry optimization, can be easily identified and corrected in practice via the analysis of the numerous ss-NMR experimental parameters that are sensitive to the interactions experienced by ^1^H nuclei. 

## 3. To Transfer or Not to Transfer

Controlling molecular assembly via crystal engineering represents an effective way to modify the physical properties of organic solids [88]. This approach has been most widely used on pharmaceuticals [89,90,91] to tailor properties like solubility, dissolution rate, hygroscopicity, or morphology, but attempts to engineer other materials, for instance to tune optical properties [92,93,94,95,96], thermochromism [97], or to improve the detonation performance vs. sensitivity to external stimuli [98] in high-energy-density molecular crystals by cocrystallization are also reported. One route to modify properties is the formation of multicomponent complexes, typically acid–base pairs, where the introduction of a second molecule into the crystal lattice can facilitate the engineering of solid-state forms with significantly different properties [99,100,101] via the modulation of the intermolecular noncovalent interactions. If cocrystals are formed, both components are neutral and interact through designable interactions such as hydrogen or halogen bonding. Salts are usually formed by intermolecular proton transfer, modifying the nature of the noncovalent interactions by allowing for charge-assisted hydrogen bonding. Acid and base groups within the same molecule can also be exploited to introduce electrostatic interactions by proton transfer, which creates zwitterionic molecular crystals [102].

Rational design of new organic materials by crystal engineering requires the accumulation of a large amount of data and knowledge with relevance for building up a consistent set of structure–property relationships. Based on such kind of knowledge, ideally, materials with desired properties for various applications can be engineered from adequate building blocks. Along this line, if charge assisted hydrogen bonding is required to be introduced in a multicomponent system by proton transfer, that is, to obtain a salt, a first empirical rule that can be used is based upon the relative strength of the acid and base pair, the so-called “rule of 3”. For values of ΔpK_a_ = pK_a_ (protonated base) −pK_a_ (acid) less than 0 a cocrystal forms, while for differences greater than 3, a molecular salt forms [103,104]. However, the ΔpK_a_ parameter is insufficient for predicting salt–cocrystal formation in the solid state [105] when ΔpK_a_ is between 0 and 3. This relatively narrow interface region, often named the salt–cocrystal continuum, poses a difficulty to crystal engineering approaches targeting specific supramolecular assembly, but also represents an opportunity to explore uncharted territory. Operating within this region, either charged or neutral states can be obtained, which, given the typically very different physical properties of ionic salts and neutral cocrystals, presents opportunities for property tuning. The salt–cocrystal continuum has been the subject of many studies [106,107] and confirmed to hold true in all of them. However, no other molecular properties were identified so far that could serve as predictors for proton transfer in salts or zwitterionic solids. In such cases, it was found that the crystalline environment, in particular the position of the shared hydrogen atom, plays a major role in establishing whether proton transfer can, or cannot, occur. 

Therefore, one major issue for accurately characterizing solids in the salt–cocrystal continuum is to identify parameters with high sensitivity to proton location. Given the low sensitivity of X-ray diffraction to hydrogen, other structural parameters related to the donor and/or acceptor atoms have been considered for this purpose. For instance, the C–O and C–N bond lengths, or C–N–C bond angle, have been found as good indicators of proton transfer involving carboxyl and amine moieties [108,109]. Moreover, spectroscopic parameters like characteristic IR vibration frequencies [110], ss-NMR chemical shifts [82,111], and XPS absorption peaks associated with such donor/acceptor groups [111] provide useful complementary information, often, when working on powders, with increased reliability compared to that offered by X-ray determined structural parameters. In this context, NMR crystallography emerged as an ideal tool for identifying and characterizing proton transfer in organic solids. It combines information from the analysis of X-ray and ss-NMR experimental data, with the DFT computations providing the necessary link between them: the final result of such an approach is a refined crystal structure solution, where the labile hydrogens are expected to be spatially constrained with superior accuracy compared to what could be achieved by each of these methods alone. In conclusion, structural parameters like bond lengths and bond angles around the donor/acceptor sites, as well as spectroscopic parameters of the involved atoms/nuclei are already well-established indicators for proton transfer in organic solids. The two examples discussed in this section perfectly illustrates, on the one hand, that both types of parameters are easily accessible by specific NMR crystallography operations, with the effect of increasing the confidence level in the obtained structure, but, on the other hand, that the strategies followed for their determination may be very diverse, depending on the nature of the sample and the complexity of the molecule(s) in the lattice.

**Figure 2:**
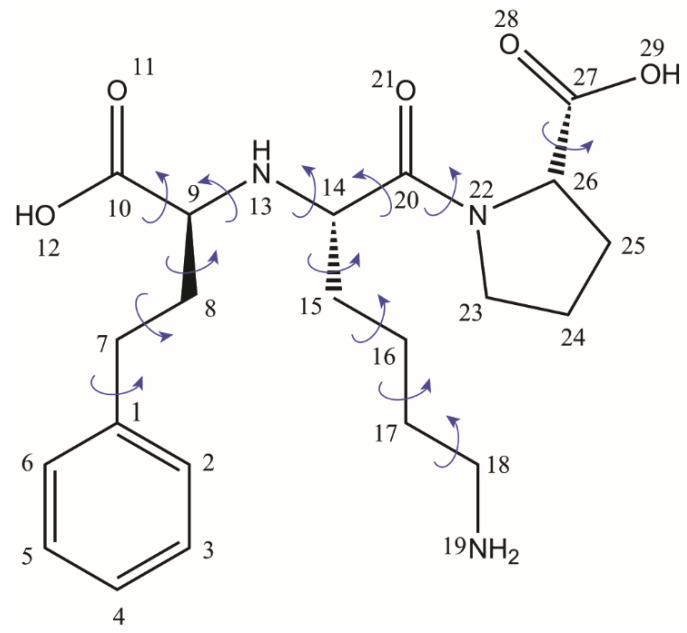
The chemical structure of Lisinopril, which emphasizes the flexible torsion angles considered for powder X-ray diffraction (PXRD) structure determination.

The first example is that of the active pharmaceutical ingredient Lisinopril, an Angiotensin Converting Enzyme (ACE) inhibitor, widely used for hypertension and heart failure [112]. The most stable crystalline form of Lisinopril found in commercial drugs is a dihydrate, but less stable anhydrous and metastable monohydrate forms were also reported [113,114]. What makes this case interesting is that, despite of being on the market since 1987, the crystal structure of Lisinopril dihydrate could not be solved until recently when, within a time span of only one year, three crystal structure models were reported using completely different approaches: the first crystal structure was determined by Uekusa et al. by PXRD using synchrotron X-ray sources [113], next, growing a single crystal of sufficient size has been finally succeeded by the group of Caira, so that a single-crystal X-Ray structure could be determined [114], and then, almost simultaneously, our group published a crystal structure solved by NMR crystallography on powder, by using conventional laboratory X-Ray sources [59]. This rather long story of the Lisinopril dihydrate crystal structure determination is explained, on the one hand, by the difficulty of finding the proper conditions to grow it as a single crystal and, on the other hand, by the complexity of the Lisionopril molecule, with 13 flexible torsion angles, see Figure 2, which renders its structural determination by conventional PXRD methods alone almost impossible. However, the fact that the efforts to overcome the obstacles along of both these lines have been rewarded in the end with three different crystal structure models is very useful in the context of the present discussion, because one can directly assess the progress reached by NMR crystallography in structural characterization of organic solids, even though this approach incorporates more ordinary analytical tools than is the case of synchrotron and single-crystal X-Ray diffraction. 

In the following, we compare these three structural models in terms of all the relevant parameters, with special attention being paid to how the problem of locating the labile hydrogen atoms was treated in each case. For simplicity, we name the models according to the particular technique employed for their determination: Lisi-PXRD-Synch, Lisi-SC-XRD, and Lisi-NMR-Cryst, respectively. The unit cell parameters were found very close to each other, with only a small reduction of ~1.2% in the unit cell volume determined on single crystal relative to the values obtained from powder. This contraction can be reasonably explained by the fact that the single crystal X-ray pattern was measured at low temperature (173 K), whereas the powder patterns were recorded at room temperature. The conclusion is further supported by the unit cell parameters obtained from the PXRD data analysis, which were almost identical in the both cases, of synchrotron and laboratory X-ray sources. 

**Figure 3:**
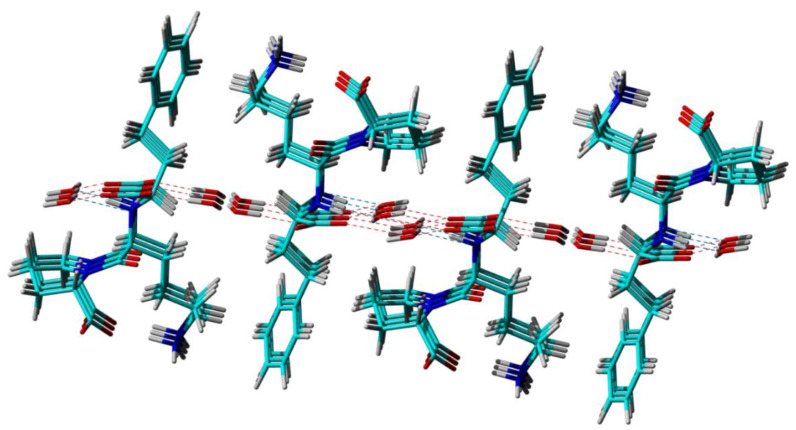
Crystal packing of Lisinopril dihydrate, highlighting the formation of two water channels and the hydrogen bonding networks that keep the water molecules inside them.

Furthermore, from the perspective of long-range ordering, the three different techniques reveal basically the same crystal packing patterns, offering an identical picture of the way that the Lisinopril and water molecules arrange themselves relative to each other in the lattice to form two distinct water channels along the *b* crystallographic axis, see Figure 3. Differences, however, exist at the level of local structural features like bond lengths, bond angles, and local conformations (torsion angles), which stems from the different ways that molecular structures in solid are built up from the analysis of the experimental data: diffraction patterns in the case of the Lisi-PXRD-Synch and Lisi-SC-XRD models, and combined diffraction, ss-NMR and DFT computations for Lisi-NMR-Cryst, respectively. As mentioned in [114], the major limitations of the procedures based on PXRD data is the need to restrain molecular parameters to some “standard” values during the various stages of model refinement, which is not the case of single-crystal X-Ray analysis, where unbiased optimization of atoms position is permitted by the overdetermination of the ratio between the amount of the available data and number of the parameters to be optimized. Therefore, the Lisi-SC-XRD crystal structure, which is expected to best approach the real crystal structure of Lisinopril dihydrate, is taken here as a reference model for discussing the solutions provided by the other two powder-based approaches. 

The bond lengths and bond angles of Lisinopril molecule in a PXRD crystal structure model essentially depend on the molecular structure taken as input in the first refinement step, i.e., the search for an approximate structure solution, and also on the procedure used to optimize these parameters during further refinement. In the case of Lisi-PXRD-Synch, the starting molecular structure was imported from the crystal structure of a Cu complex of Lisionopril [115], the only single-crystal structure of a related compound available at that time. Only 13 flexible torsion angles were optimized when searching for the approximate solution, whereas all the atomic coordinates, implicitly, also the bond lengths and angles, were considered for the final Rietveldt refinement. The hydrogen atoms were placed at standard position relative to the chemically bonded heteroatoms. A different approach has been considered by us for deriving the Lisi-NMR-Cryst model [59]. The input Lisinopril molecular structure was optimized at the molecular mechanics level of theory, and only the flexible torsion angles were adjusted during both the search for an approximate structure solution and Rietveldt refinement steps. This means that in the Rietveldt refined model the bond lengths and bond angles had the same approximate values as in the input molecule. Their fine adjustment towards more realistic values was achieved during the DFT geometry optimization step, when, from the considered PXRD candidate structures, in the end we were able to select the crystal structure solution which simultaneously satisfies the following two conditions; (i) the lowest lattice energy and (ii) shows the best fit between the computed and measured ^15^N, ^13^C, and ^1^H ss-NMR chemical shifts. To compensate for the increase in the R_wp_ fit parameter observed after the readjustment in the bond lengths and angles, Rietveld refinement was applied once again over selectively reintroduced groups of degrees of freedom: the best solution, leading to the Lisi-NMR-Cryst model, was obtained when changing the water molecules positions within the two water channels. 

**Figure 4:**
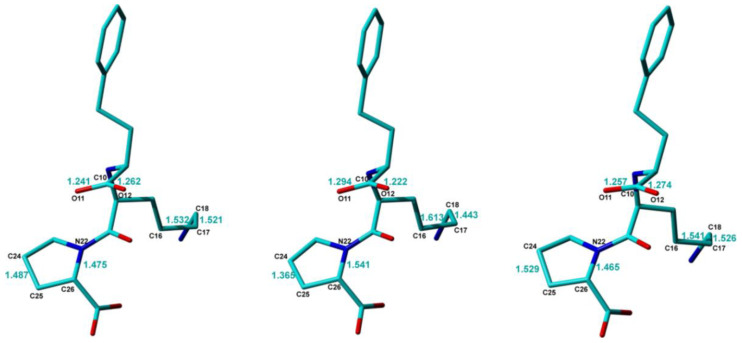
Comparison between the Lisinopril molecular conformations in the three analyzed models, Lisi-SC-XRD (**left**), Lisi-PXRD-Synch (**middle**), and Lisi-NMR-Cryst (**right**). The C–C and C–N bond lengths with differences larger than 0.05 Å, and the C–O lengths within the -C9-OO carboxyl group are highlighted.

The conformations of the Lisinopril molecule in the three crystal structure models are very close to each other, with RMSD values less than 0.1 Å between any pair of molecules, except for some notable differences which are highlighted in Figure 4. One of them refers to the difference between the two C–O bond lengths within the -C10-OO carboxyl moiety, which is significantly larger, ~0.07 Å, in Lisi-PXRD-Synch than the value of only ~0.02 Å obtained in the other two models. Possibly to compensate for this difference, the C15–C16 bond of 1.61 Å and the C16–C17 bond of 1.44 Å, determined in the Lisi-PXRD-Synch structure, fall outside the 1.50 to 1.53 Å range, which is typical for CH_2_-CH_2_ bond-lengths. By contrast, both the C21-O bonds and these two C-C bonds, obtained in the case of Lisi-SC-XRD and Lisi-NMR-Cryst models, are more consistent with each other, see Figure 4. Moreover, the C24-C25 and N22-C26 bonds within the pyrrolidine ring have closer values in Lisi-SC-XRD and Lisi-NMR-Cryst and significantly different to the lengths determined in Lisi-PXRD-Synch, which again fall outside the typical range characteristic for this type of chemical bonds.

At the end of this comparative analysis, we discuss the procedures used to locate the hydrogen atoms within the three distinct approaches, with the main focus on the labile hydrogens belonging to the -C10OOH and -C27OOH carboxylic acid groups in the original isolated Lisinopril molecule. In the case of Lisi-SC-XRD model, their positions have been determined by using the standard procedure of difference Fourier map, with a special attention being paid to the potential zwitterionic nature of the Lisionopril molecule. This was confirmed actually for the both amino groups, with electron density peaks corresponding to the -N13H_2_^+^ and -N19H_3_^+^ moieties when the Lisinopril molecule is packed in the dihydrate crystal lattice. Accordingly, no electron densities assignable as hydrogen atoms were found in the vicinity of the -C10OO^−^ and -C27OO^−^ groups, which thus transform to carboxylate ions. In fact, these proton transfer processes could be anticipated based on the determined positions of the bonded heteroatoms, in particular from the values of the corresponding C-O distances, and the C9-N13-C14 bond angle, which, as mentioned previously, can be taken as good local structural indicators of proton transfer. 

The double zwitterionic character of Lisinopril in its dihydrate crystalline form was correctly determined also in the case of the Lisi-NMR-Cryst model, i.e., when employing the NMR crystallography approach. The proton transfer from the carboxyl to the amine groups was obtained there only after the DFT geometry optimization was completed, because, until that step in the refinement process the hydrogen atoms position relative to the bonded heteroatoms were kept fixed to their values taken in the input structure of the Lisinopril molecule. By contrast, only the -C27OO^−^ → -N19H_3_^+^ proton transfer could be identified in Lisi-PXRD-Synch: for this, the empirical rule based upon the relative strength of the acid and base pair was used, in particular the fact that ΔpK_a_ > 6 between such moieties in amino acids [116]. A different argument was applied to the -C10OOH/-N13H acid–base pair. Combined with the fact that no significant rearrangements in the corresponding C-O bond lengths were obtained after Rietveld refinement, the acidic character of this carboxyl group was considered unchanged in the reported crystal structure solution [113]. 

Finally, it is important to elaborate a little bit more on the practical significance of the above discussed results. Single-crystal X-ray diffraction, obviously the golden standard technique in crystal structure determination, is generally very successful in identifying proton transfer. This can be done either directly from the electron densities that are the most realistically assignable to hydrogen atoms, or indirectly, from local structural parameters characteristic to the donor/acceptor atoms—the latter can be located with an even increased accuracy than the hydrogens. However, not every organic solid can be grown as a single crystal suitable for X-ray diffraction studies. For others, this is very difficult to achieve, with the case presented here being an illustrative example. In fact, before finding the proper crystallization conditions for Lisinopril dihydrate [114], Lisi-PXRD-Synch has been the only reported crystal structure model, which proved erroneous in providing the real ionization state of the Lisinopril molecule. Thus, complex molecules with a large number of degrees of freedom to be refined during crystal structure determination may still pose serious obstacles for accurately locating the hydrogen atoms from powders even under high-resolution conditions achievable with synchrotron X-ray sources. We assume here that this can happen by an “error compensation” effect, i.e., small errors in determining the real position of certain atoms could compensate for each other in the simulated PXRD pattern, eventually leading to a very good fit of the experimental pattern. Examples for such small errors in the case of Lisi-PXRD-Synch model are the bond-lengths highlighted in Figure 4: the fact that these errors affect also the position of the oxygen atoms in -C10OOH carboxyl moiety, clearly explains why the proton transfer to -N13H amine could not be predicted based on the corresponding C–O bond lengths. 

**Figure 5:**
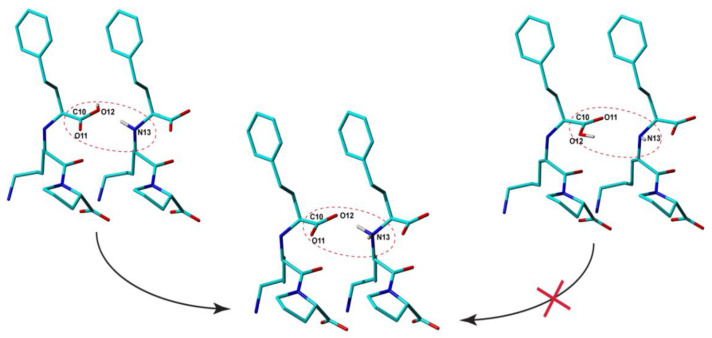
Intermolecular arrangements of Lisinopril molecules, which differ between themselves by the relative conformations of the -C10OOH and -N13H amine moieties: by Density Functional Theory (DFT) geometry optimization of the corresponding crystal structure models, one of them leads to proton transfer, whereas the other does not; see the text for details.

From this perspective, the situation might appear even worse when NMR crystallography is employed: the Rietveldt refined structures obviously contain in this case more errors in the bond lengths and bond angles than Lisi-PXRD-Synch, including the ionization state, because all these parameters have been constrained to their approximate values assigned to the input Lisinopril molecular structure and kept constant throughout the entire PXRD data analysis. Moreover, it is also characterized by ambiguities in the provided results, generally giving rise to multiple candidate structure solutions. However, it is exactly this ambiguity which is advantageous for the NMR crystallography approach, because it allows us to better explore the available conformational space, and thus increase the chance to find the conformation closest to that found in the real compound. By DFT geometry optimization, this particular conformation is expected to lead to the structure with the lowest lattice energy and also with the best fit of the relevant experimental ss-NMR parameters. In the Lisinopril dihydrate case, ambiguities were obtained with respect to the conformation of the carboxyl groups: for each of them, two different conformations flipped with 180⁰ around the corresponding -C-COOH bond were found almost equivalent with respect to the R_wp_ fit parameter of the X-Ray diffraction pattern; this is illustrated in Figure 5 for the -C10OOH carboxyl moiety. Therefore, all these candidate structures have been subjected to DFT geometry optimization. Then, the DFT refined crystal structures have been ranked according to the lattice energy and the quality of the fit between the calculated and measured ^13^C, ^15^N, and ^1^H chemical shifts. Finally, the Lisi-NMR-Cryst model, which correctly predicts the formation of the two carboxylate ions by proton transfer to the neighboring amine moieties, was found to best satisfy the both conditions. 

**Figure 6:**
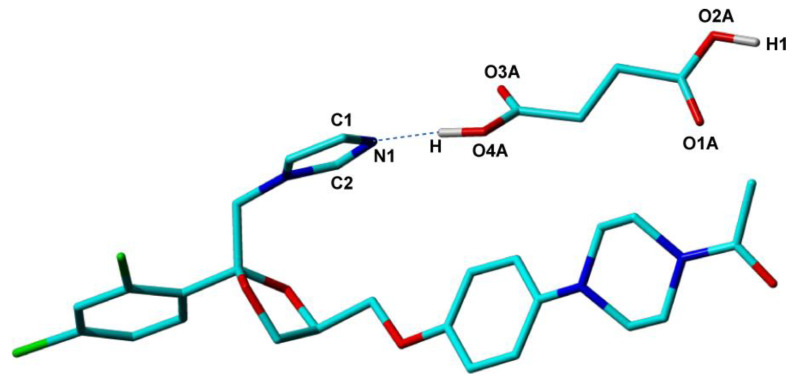
The O4A-H … N1 hydrogen bond between ketoconazole and succinic acid molecules in crystalline structure of the formed cocrystal.

The second example is that of the ketoconazole–succinic acid binary compound (KET-SucA), in 1:1 stoichiometry. It belongs to a series of four new solid forms of KET with dicarboxylic acids (oxalic, fumaric, succinic, and adipic) obtained by our group in a study aimed at improving solubility compared to that of original KET molecule. This new solid form turned out in the end to have solubility 75 times as large as that of ketoconazole [82], the second largest in the whole series. The crystal structures of all these new compounds were determined by single-crystal X-ray diffraction with good accuracy. However, for KET-SucA, the location of the hydroxyl H atom—H(O4A) in Figure 6-which determines the ionization state of the compound, could not be determined from the difference Fourier map, most probably due to increased disorder around this molecular site. The ΔpK_a_ difference of 2.31 between KET and SucA, places this binary compound in the salt–cocrystal continuum, so that an estimation of the ionization state based on the ΔpK_a_ predictor was not possible. Instead, a neutral ionization state of KET-SucA, thus a cocrystal solid form, could be anticipated based on local structural parameters around the O4A-H hydroxyl of succinic acid and N1 nitrogen of ketoconazole. The distance d(C4A−O3A) = 1.182 Å smaller than d(C4A−O4A) = 1.264 Å (a difference of about 0.918 Å), indicate a double bond for the first, and a single bond for the second one, specific to a neutral carboxylic group [117]. It is also known that the C−N−C angle in N-heterocycles is sensitive to the protonation of nitrogen, having in general values smaller in neutral solid forms than in protonated forms [109,117]. In the case of imidazole ring, for instance, in histidine, Malinski and her co-workers [118] found values of 108° ÷ 109° for protonated states. In the determined crystal structure of the KET–succinic acid solid form, the C−N−C angle value is 105.99°, smaller than that of KET-oxalate salt (109.12°) and similar to KET co-crystals with fumaric (105.69°) and adipic acid (105.15°), indicating also a neutral state for KET-SucA. 

To further verify the conclusion derived from the values of the bond lengths and bond angles around the O4A-H hydrogen, an NMR crystallography analysis has been also performed. First, the DFT computed lattice energy on the single-crystal structure of KET-SucA was shown to have a smaller value in the case when H is bonded to the O4A, with 0.82 Å bond length, than in the case when this hydrogen atom is bonded to N1, with a bond length of 0.86 Å [82]. Furthermore, when the optimization of all H atoms positions (heavy atoms being fixed to their positions determined by single-crystal X-ray diffraction) was performed on these two structural models, with the H atom bonded either to O4A or to N1, the computations converged to a unique structure, with H bonded to O4A. Both results are thus consistent with the co-crystal form of KET-SucA. For completeness, these types of calculations have been done also for the other three KET solid forms in the series, and the results were in agreement with the ionization states obtained from the single-crystal X-ray analysis. Finally, ss-NMR experimental validation was achieved by comparing the computed and measured ^15^N chemical shifts. This experimental parameter has provided with excellent sensitivity the ultimate proof that, among all the studied binary compounds of ketoconazole, only KET-oxalate leads to proton transfer to the N1 nitrogen: its ^15^N ss-NMR line was found shifted with more than 50 ppm relative to the N1 line in the spectra of the other three compounds, including KET-succA, both in calculations and in practice [82]. 

## 4. With or without Transfer, There Is Definitely Sharing

Proton transfer occurs in organic solids when labile hydrogens are energetically more favorable to “migrate” from acid to base groups. However, no matter if the transfer occurs or not, that particular labile proton is actually shared between the donor and acceptor atoms in the acid–base pair, in either case forming a hydrogen bond which may significantly contribute to the global energetics of the resulting crystalline solid. Put in this broader context, the requirement for accurate localization of labile hydrogen atoms is important mostly because it offers an opportunity to characterize the relative strengths of the formed hydrogen bonds with respect to other noncovalent interactions, which gives the basis for explaining various macroscopic properties of the material. In the examples above, the detection of proton transfer processes appeared as a consequence of determining the correct geometry of the underlying hydrogen bonds: in an NMR crystallography approach, this essentially depends on the possibility of reaching the absolute minimum in the potential energy surface during the final DFT geometry optimization of the PXRD crystal structure model. To maximize this chance, one route is to generate multiple PXRD trial structures by changing local structural parameters around that particular donor–acceptor pair, however, provided that the positions of the other non-hydrogen atoms have been constrained with sufficient accuracy by Rietveld refinement. In the case of Lisinopril dihydrate these local parameters referred to the conformation of the carboxyl groups around the adjacent C–C bond, whereas for the ketoconazole–dicarboxylic acids binary systems the variable structural parameter was taken the position of the shared proton in between the donor and acceptor sites. 

**Figure 7:**
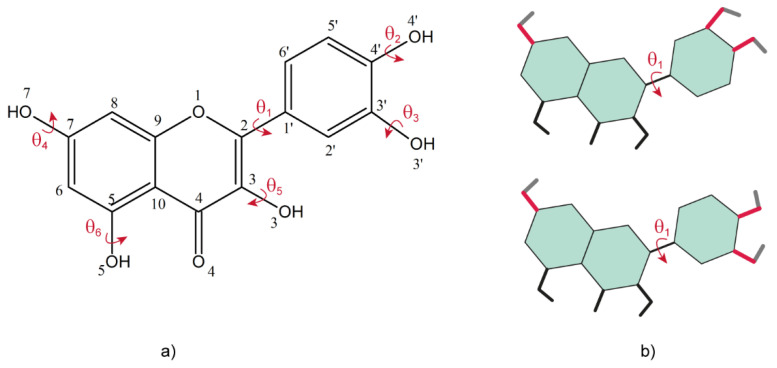
(**a**) The chemical structure of Quercetin, which emphasizes the flexible torsion angles considered for structure determination, including DFT geometry optimization; (**b**) the two distinct relative conformations of the catechol and benzopyran rings found in all the reported solid forms of Quercetin, close to syn—bottom, and close to anti—up.

In the following, we show that the same general considerations apply also when characterizing any hydrogen bond that may form in organic solids, whether it implies proton transfer or not. We illustrate this statement with the example of various solid forms of Quercetin, a naturally occurring flavonoid intensively studied in our group [84,85,86,87]. As can be seen from the molecular structure of Quercetin, the crystal structure determination from powder should not be a difficult task, because there is only one flexible torsion angle, denoted by θ_1_ in Figure 7a, that must be adjusted for structure refinement. This angle defines the relative orientation between the catechol and benzopyran rings: in all the crystal structures of the Quercetin solid forms reported so far θ_1_ was found either close to syn, or close to anti, conformations of the two rings, defined with respect to perfect planarity of the whole molecule, see Figure 7b. The lowest energy of the isolated Quercetin molecule was obtained for θ_1_ ~30° [84], thus close to the *syn* conformation: the fact that in many Quercetin solid forms this torsion angle is flipped with up to 120°, approaching the anti-conformation, is an effect of the intermolecular interactions with the neighboring molecules in the lattice, most probably dominated by the multiple hydrogen bonds that are likely to be formed by its numerous hydroxyl groups.

The value of the θ_1_ torsion angle can be determined with very good accuracy from the X-Ray data analysis, even on powders. Moreover, ss-NMR is very sensitive to distinguish between different conformations around this angle, in particular the C2′ and C6′ ^13^C chemical shifts have almost the same values in Quercetin dihydrate (where θ_1_ corresponds to a close to *anti* conformation), whereas they are separated by about 14 ppm in anhydrous Quercetin, where the two rings adopt a close to *syn* conformation. The other five flexible torsion angles, θ_2_–θ_6_, are related with the spatial orientation of the corresponding hydroxyl groups (Figure 7a) and are therefore undetectable by PXRD. Two of them, θ_5_ and θ_6_, cannot take arbitrary values. The former is related to the orientation of the -O5H hydroxyl, which was shown both theoretically and experimentally that will always form a strong intramolecular hydrogen bond with the O4 oxygen atom. Indicative for this bond is the peak with chemical shifts larger than 13 ppm, well separated from the all other NMR lines, which is obtained in the ^1^H ss-NMR of all the studied Quercetin solid forms. The latter also shows a tendency of the -O3H hydroxyl group to orient towards the O4 oxygen, although with larger deviations with respect to the benzopyran ring plane than the -O4H hydroxyl. Most probably, this off-plane deviation comes from the fact that the O3H hydrogen is involved in bonding with both, O4 and other oxygen acceptors from neighboring molecules.

Besides the θ_5_ and θ_6_ torsion angles, of which values are partially constrained by the O5-H…O4 and O3-H…O4 intramolecular hydrogen interactions, the other three hydroxyl groups can adopt any orientation in the resulting compound, depending on the direction and distance to the closest acceptor atom in the crystal lattice. Therefore, the probability of not obtaining in the end the correct hydrogen bonding pattern by DFT geometry optimization of the Rietveld refined structure model if starting from arbitrary values of the θ_2_–θ_4_ torsion angles is the same as discussed above in the case of Lisinopril dihydrate. The risk is that the structure might be trapped in a local minimum of the potential energy surface in the course of computations if the starting conformation of these hydroxyl groups are far off their real conformation, as was also observed for the two carboxyl groups in Lisinopril. This problem was addressed in our work on anhydrous quercetin [84] by deriving three distinct approximate crystal structure solutions, each of which being built upon input quercetin molecules with structures established based on very different considerations—step two in the general NMR crystallography workflow depicted in Figure 1. Consequently, the complete crystal structure determination procedure was repeated three times, each search run producing its own approximate solution, which was next submitted to Rietveldt refinement. The crystal structure models obtained in the end, named Quer-1, Quer-2, and Quer-3, were subjected to DFT geometry optimization, first with respect to only the hydrogen atom positions, then also over the all atom positions, and finally ranked according to the criteria thoroughly discussed above. Only the first two models are illustrative for the discussion here and, therefore, presented in more details. 

**Figure 8:**
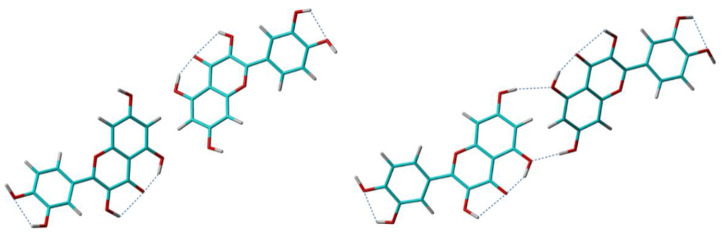
Intermolecular contacts of Quercetin molecules along the benzopyran rings depending on the conformation of the –O7-H hydroxyl in two crystal structure models of anhydrous Quercetin, Quer-1 (**left**), and Quer-2 (**right**).

The Quer-1 model was obtained by following the quickest but, at the same time, the roughest approach: the input quercetin molecule was drawn from scratch and its structure optimized at the lowest approximation level, i.e., by molecular mechanics (MM) calculations. This leaves all the degrees of freedom to be refined at later stages: the molecular position within the unit cell and the θ_1_ torsion angle, during the search for the approximate solution and Rietveldt refinement, whereas the bond lengths, bond angles, and the other torsion angles, including the conformations of the hydroxyl groups, during the two DFT geometry optimization stages. Notably, the strong intramolecular hydrogen bonding between -O5H hydroxyl and the O4 oxygen atom was already identified during the MM structure optimization of the isolated quercetin molecule, so that the correct value of the θ_6_ torsion angle was set at the beginning of all the refinement steps, and left almost unchanged until the end of crystal structure determination process. After DFT geometry optimization, two of the other four hydroxyl conformations in solid were modified with respect to their values in the isolated molecule, to accommodate the formation of the intermolecular hydrogen bonding pattern depicted in Figure 8. 

The second model, Quer-2, was derived following an opposite approach: except θ_6_, of which value is constrained by the O5-H⋯O4 hydrogen bond, the input molecular structures were obtained by a systematic grid search over all the remaining θ flexible torsion angles, that generated in the end about 250,000 distinct conformations of the isolated quercetin molecule. Confining to thermal energy at room temperature, only three of these structures had to be employed as input for subsequent XRPD analysis and DFT geometry optimization. Finally, the crystal structure model with the lowest lattice energy, Quer-2, was selected as the output of this crystal structure determination approach. Quer-2 has also lower lattice energy compared to Quer-1, which is not surprising given the fact that it was built on larger number of possible initial conformations of the input quercetin molecule. As often mentioned before, this significantly reduces the risk to have all the refined crystal structures trapped in a local minimum of the potential energy surface during DFT geometry optimization, and thus increases the chances to reach the absolute minimum energy for one of the considered trial models. 

What was less expected was the rather large energy difference of 25.8 kcal/mol between the two crystal structure solutions. A close inspection of the formed crystal packing patterns reveals the source of this difference: whereas the positions of the quercetin molecules within the unit cell and the molecular degrees of freedom related to non-hydrogen atoms were refined to almost identical values at the end of crystal structure determination process in two cases, the O7-H hydroxyl was driven in an opposite conformation in Quer-1 compared to that in Quer-2. This leads to the missing of the O7-H⋯O5 intermolecular hydrogen bond in Quer-1 (see Figure 8): since there are four such hydrogen bonds in the unit cell, the calculated energy difference of 25.8 kcal/mol between the two crystal structure models is equivalent with an energy of 6.45 kcal/mol stored in the O7-H⋯O5 hydrogen bond, which is fully consistent with its geometrical parameters. As customary in NMR crystallography, these theoretical findings were finally verified with respect to the measured ss-NMR spectral parameters, leading basically to the same conclusion: Quer-2 represents the best model for the real crystal structure of anhydrous quercetin [84]. The main arguments for this conclusion were that, (i) large deviations between the experimental and computed ^13^C chemical shifts were found for the C8 and C5 in Quer-1, thus exactly the carbon sites mostly affected by intra- and intermolecular contacts with the O7-H hydroxyl group, and (ii), a cross-peak in the 2D ^1^H-^1^H double-quantum ss-NMR spectrum of anhydrous quercetin predicted on the basis of the O7-H conformation in Quer-1 was not obtained experimentally. 

Although a significant improvement in accuracy was obtained by the approach leading to the Quer-2 model, this is neither exhaustive (only three out of the eight realistically possible hydroxyl conformations related to the θ_2_–θ_4_ torsion angles were selected for the staring quercetin molecule), nor very efficient (all-atoms DFT geometry optimization, which is the most time-consuming step in the crystal structure determination process, must be repeated for all the selected trial models). A more rational approach was introduced in a subsequent work [85], by which both drawbacks could be removed. The entire set of eight different relative conformations of the O4′-H, O3′-H, and O7-H hydroxyls were considered in the analysis, however, this time not for the starting structures of the isolated quercetin molecule, but for the Rietveld refined PXRD crystal structure solution. Each of these eight different crystal structures was next subjected to only H-atoms DFT geometry optimization and the resulting structures were ranked according to their lattice energy. Only the model with the lowest energy is considered finally for all-atoms DFT geometry optimization, which leads also to the desired computational time savings. The reliability of the new approach was demonstrated [85] on quercetin dihydrate and anhydrous quercetin, two solid forms with known crystal structure, and then also successfully applied to the crystal structure determination of other new quercetin solid forms [86,87].

## 5. Conclusions and Outlook

The present work reviews recent achievements of NMR crystallography in the characterization of crystalline organic solids, with the main emphasis being put on the specific tools developed with the purpose of locating the labile hydrogen atoms with increasing precision in between donor and acceptor sites. The problem is relevant in the context of the key role that such hydrogen atoms generally play in the global energetics of the lattice, often determining the established supramolecular arrangements and crystal packing patterns through the formation of complex hydrogen bonding networks. Some of these achievements are illustrated with examples from our group past work on crystal structure determination by NMR crystallography: the examples are chosen so as to be representative for the problem being addressed here and all the results are discussed and reinterpreted only from the narrow perspective of labile proton location within the solved crystal structures. Although each molecular crystal may have its own specificities in this respect, the presented case studies also highlight certain aspects that should be generally considered, irrespective to the investigated system:

(1) The labile protons localization is a meaningful process only if the positions of the non-hydrogen atoms within the unit cell are constrained with sufficient accuracy from the analysis of the XRD data. On powders, NMR crystallography offers powerful indicators (e.g., ^13^C/^15^N chemical shifts) to assess the compliance with this requirement. 

(2) To minimize the risk of the structure being trapped in a local minimum of the potential energy surface during DFT geometry optimization, a set of multiple trial models should be considered, which results from taking into account all the realistically possible conformations of the chemical moieties containing the unconstrained labile protons.

(3) The finally selected crystal structure solution should simultaneously satisfy a series of conditions: has the lowest lattice energy, provides the best fit between the computed and measured ss-NMR parameters and, optionally, is leading to the most realistic hydrogen bonding pattern. 

Within this general methodology, obviously, the geometry optimization of the Rietveld refined crystal structure by quantum chemistry calculations in extended periodic systems plays a central role, because the local geometry around labile hydrogen atoms is ultimately established by such computations. The CASTEP implementation of DFT computations based on the GIPAW method has become almost a golden standard in the field, so that further progresses are expected from continuing upgrades to its various components. In particular, the fact that convergence is defined with respect to the forces acting upon each atom represents the main weakness, being actually responsible for the fact that sometimes the relaxed structure is driven to a local minimum in the potential energy surface and not to the absolute minimum, i.e., not towards the actual crystal structure of the compound. Therefore, finding solutions to this problem would represent an important breakthrough for the final DFT refinement step in the whole structure determination process. Besides making DFT geometry optimization more robust with respect to the inherent errors incorporated into PXRD crystal structures, there is also need for improvements in the accuracy of the DFT computed ss-NMR parameters: if isotropic chemical shifts can now be calculated quite precisely, with typical RMSD values of 2 ppm for ^13^C, or 0.5 ppm for ^1^H nuclei, and show weak dependence on temperature, the principal components of the corresponding chemical shielding tensors show increased sensitivity to temperature effects. Thus, progresses in more efficiently accounting for vibrational and long range dispersion effects are expected to reduce not only the still large errors in the computed chemical shielding tensor parameters, but also in other interaction parameters like the hydrogen-mediated J-couplings, or ^17^O quadrupolar interaction tensors, if is to mention only the most relevant for the present discussion.
